# Social Statuses, Quality of Social Relationships, and Self-Esteem Among Older Adults in Canada: Resource Multiplication or Substitution?

**DOI:** 10.1177/07334648241253729

**Published:** 2024-05-28

**Authors:** Megan Harmon, Alex Bierman, Yeonjung Lee

**Affiliations:** 1 Department of Community Health Sciences, 2129 University of Calgary, Calgary, AB, Canada; 2 Department of Sociology, University of Calgary, Calgary, AB, Canada; 3 Faculty of Social Work, 2129 University of Calgary, Calgary, AB, Canada; 4 School of Social Welfare, Chung-Ang University, Seoul, Korea

**Keywords:** self-esteem, education, gender, social relationships

## Abstract

This research examines how older adults’ self-esteem is shaped by core social statuses and ongoing social relationships. Based on a national survey of Canadian older adults (*N* = 4010), analyses show that men have greater self-esteem than women, as do people with higher quality of social relationships and a high degree of educational attainment. Neither gender nor quality of social relationships intersect with education to shape self-esteem, but quality of social relationships is more strongly associated with self-esteem for women. Consequently, strong gender differences are observed at low levels of relationship quality, but these gender differences are negated at high levels of relationship quality. This research shows that social statuses and relationships cohere to shape self-esteem in later-life, but gender differences are not evident in the context of high-quality social relationships. Conversely, educational attainment appears to be a key determinant of high self-esteem, irrespective of gender or quality of social relationships.

**What this paper adds**

• As individuals age, women and individuals with lower levels of education may be at an increased mental health risk due to lower levels of self-esteem.
• Advantages in self-esteem due to high levels of educational attainment do not differ by gender.
• High-quality social relationships not only benefit the self-esteem of older adults but can also negate gender differences in self-esteem.

**Applications of study findings**

• Programs which focus on helping older adults to cultivate and maintain high-quality social relationships are likely to be critical for reinforcing self-esteem, which is in turn likely to have substantial mental health benefits.
• These programs are likely to be especially important for erasing the gender gap in self-esteem in later-life and offsetting disadvantages in self-esteem faced by older adults with low levels of education.

How do fundamental social statuses and social resources independently and jointly contribute to self-esteem in later-life? This is a critical question because self-esteem is an important resource for both mental and physical health among older adults (Krause, 2016). According to Pearlin’s (1999) stress process model (SPM), each aspect of the stress process is shaped by fundamental statuses, with gender and education of focal interest as core social statuses that condition this process (Lee & Bierman, 2019; Milkie et al., 2008). The SPM in turn identifies self-esteem as a core resource for mental health that will be shaped by these social statuses, as well as additional social resources, and their intersection (Pearlin & Bierman, 2013). Social stratification and the availability of social resources are especially salient for older adults, who often experience role loss, the loss of independence, and altered interpersonal relationships, all of which can contribute to a reduction in sense of self-worth (Orth et al., 2015). Consequently, remaining markers of advantage or social recognition are likely to be accentuated in their importance and salience as markers of individual value and worth.

Despite the importance of self-esteem for mental health in later-life, influences on self-esteem among older adults have seldom been examined in recent national studies. Less recent Canadian self-esteem research including older adults can, for example, be seen in the work of McMullin and Cairney (2004). Yet, the combination of the Great Recession and subsequent COVID-19 pandemic have led to rapid social and economic transformations (Bonotti & Zech, 2021; Danziger, 2013), leaving open a real question of whether and how social statuses and social resources continue to shape self-esteem in more recent cohorts of older adults.

In this research, we therefore use a nationally representative survey of Canadian older adults from 2021 to examine how gender, education, and the quality of older adults’ social relationships are associated with self-esteem. We not only examine the direct associations between these factors and self-esteem, but also build on work on the psychological consequences of the intersection of social statuses (Ross & Mirowsky, 2006), and examine two possible patterns for how these factors will intersect in shaping self-esteem. One is a resource multiplication pattern, in which advantaged social positions and resources compound to further increase the benefits for well-being experienced by privileged individuals (Veenstra & Vanzella-Yang, 2022). Conversely, in a resource substitution pattern, for those in disadvantaged positions, social resources compensate for each other, thereby allowing the presence of one resource to make the absence of another less damaging (Ross & Mirowsky, 2006). It is rare in research on self-esteem among older adults to examine these intersections, and the contribution of this research is therefore in not only providing recent findings on the social patterns of self-esteem among older adults, but also in showing the extent to which markers of social stratification and social resources create an interlocking social matrix of self-esteem in later-life.

## Background

Theories on the development of self-esteem can be classified into three main principles (Keith & Thompson, 2019). First, the principle of reflected appraisals suggests that self-esteem is determined by our perceptions of how we think others view us (Richard et al., 2010). According to this principle, when people perceive that they are valued and respected, self-esteem will tend to be higher (Jaret et al., 2005). Second, the principle of social comparisons suggests that when we compare ourselves to others, our self-esteem is determined by what we learn about ourselves (Keith & Thompson, 2019). According to this principle, when we compare ourselves to others, our self-evaluations are relative to a selected reference group (Corcoran, Crusius, & Mussweiler, 2011). Third, the principle of self-attributions suggests that self-esteem is determined by perceptions of personal effectiveness and competence in achieving desired outcomes (Maclean & Hill, 2015). According to this principle, when people perceive that they are effective, competent, and able to execute success in domains which they believe to be valuable, self-esteem is likely to improve (Keith & Thompson, 2019). Self-esteem can therefore be reinforced by a person’s experience in overcoming social challenges, which is a more common occurrence for those who occupy privileged statuses in society (Pearlin & Bierman, 2013). In the following section, we describe how these principles apply to the ways in which education, gender, and the quality of social relationships are likely to be associated with self-esteem in later-life.

### Education, Gender, and Their Interaction

**Figure 1.**
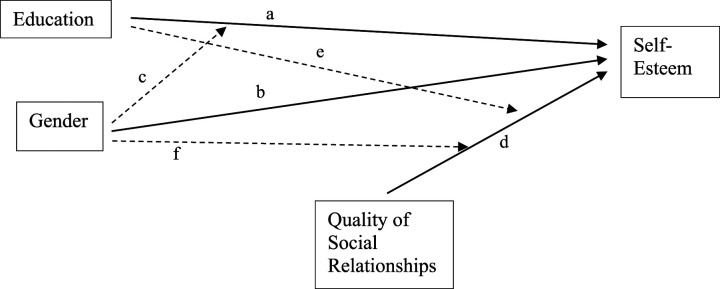
Conceptual model of focal associations with self-esteem. Solid lines indicate direct associations; dashed lines indicate moderation.

Figure 1 illustrates the focal associations under study in this research. Path A in Figure 1 demonstrates that educational attainment is expected to bolster self-esteem. Educational differences in self-esteem are expected due to the connection between self-worth and socioeconomic status (SES) (von Soest et al., 2018), with education a key component of SES (Jang et al., 2009). Higher education allows for the accumulation of human (Pelinescu, 2015) and social capital (Huang et al., 2009). The human and social capital provided by education will benefit self-esteem according to the reflected appraisals principle because individuals are able to accumulate knowledge, skills, and social ties over their lives which will tend to improve how others view them in later-life (von Soest et al., 2018). Similarly, the additional status attainment associated with jobs of higher prestige and earning potential that are facilitated by education will benefit self-esteem based on the principle of self-attributions because status attainment can increase an individual’s perception of personal effectiveness and competence in achieving their goals (Maclean & Hill, 2015). Moreover, the health and well-being that ties into education is likely to facilitate better functioning in later-life (Chiu & Wray, 2011), leading to higher self-esteem due to comparisons to similarly aged individuals with lower functioning.

Path B in Figure 1 demonstrates an expected relationship between gender and self-esteem, in which men tend to have higher self-esteem than women.^1^ Gender differences in self-esteem are expected in part because, during formative periods of self-esteem, women face greater negative reflected appraisals through sexism in classroom situations and additional gender norms that favor boys over girls (Zeigler-Hill, 2013). Additionally, during adulthood, men tend to exert higher levels of power and influence over others (Krumhuber et al., 2023; Pillemer et al., 2014). The principle of self-attributions suggests that these processes advantage self-esteem for men because a strong sense of power and influence in decision making will increase individuals’ perception of personal effectiveness and competence in achieving their goals (Guinote, 2017). Moreover, although individuals tend to experience a loss of health and changes in physical appearance as they age (Clarke & Korotchenko, 2010), women disproportionately experience the negative effects of these changes as standards of physical appearance are higher and more rigid for women than men (Åberg et al., 2020). Both a disproportionate lack of power and the loss of health and physical appearance associated with aging are in turn likely to lead to social comparisons that also deter self-esteem among women.

Yet, a stress process perspective emphasizes that different social statuses may intersect in shaping psychological resources, such as self-esteem (Pearlin & Bierman, 2013). Ross and Mirowsky (2006) expand on these ideas to outline two possible patterns by which this intersection may occur—resource multiplication and resource substitution. Resource multiplication suggests that, for those in advantaged social positions, resources compound to further increase the benefits experienced by privileged individuals (Veenstra & Vanzella-Yang, 2022). Research bears out differences in advantages of education between men and women. Across levels of education, women have lower personal financial returns (Hout, 2012), likely due in part to gender differences in balancing work and family demands (e.g., Young & Schieman, 2018). Differentials in financial returns and responses to familial demands can in turn contribute to the social disadvantages experienced by women throughout the life-course (Ridgeway, 2011). As a result, in resource multiplication, educational attainment will benefit self-esteem more for men. Alternatively, resource substitution suggests that, for those in disadvantaged positions, social resources compensate for each other, which allows the presence of one to make the absence of another less damaging (Ross & Mirowsky, 2006). From a resource substitution perspective, women tend to have fewer structural resources than men due to their disadvantaged ascribed status (Calasanti, 2010). Consequently, women may benefit more from higher education than men, as educational attainment may substitute for the structural disadvantages faced by women. Path C in Figure 1 therefore suggests an intersection between education and gender in shaping self-esteem, although we leave open a question as to whether this intersection is one of substitution or multiplication.

### Quality of Social Relationships—Intersections With Gender and Education

A stress process perspective also emphasizes that social relationships are a cornerstone of resources for mental well-being (Pearlin & Bierman, 2013), which is in line with a general understanding of self-esteem as an inherently social product that is founded and maintained within a crucible of social relationships (Serpe et al., 2019). Social relationships refer to the connections between people who have regular interactions that generate personal meaning (August & Rook, 2013). Previous research has described the quality of these relationships as composed of both positive and negative elements, such as social support and relational conflicts (Lincoln et al., 2019). The theoretical centrality of the quality of social relationships for self-esteem leads us to consider high levels of these qualities as an additional social advantage that may influence the self-esteem of older adults both independently and interactively with education and gender.

Path D in Figure 1 indicates a direct positive association between the quality of social relationships and self-esteem. According to the principle of reflected appraisals, individuals with more positive social relationships experience higher self-esteem due to a lack of aversive behaviors that evoke negative feelings toward oneself (Mavandadi et al., 2007). According to the principle of social comparisons, individuals with more positive social relationships experience higher self-esteem because these relationships provide a sense of being loved, supported, and cared for, which may buffer the self-derogation often associated with comparing oneself to those who are relatively better off (Vogel et al., 2014). According to the principle of self-attributions, those with more positive social relationships experience higher self-esteem because social support aids in adapting to stressful and challenging life events (Yıldırım & Tanrıverdi, 2021).

While much research supports the role of quality of social relationships in shaping self-esteem well into later-life (Harris & Orth, 2020), the degree to which the quality of social relationships intersects with education and gender specifically among older adults has not been as well considered. Path E in Figure 1 demonstrates the potential intersection between education and the quality of social relationships in influencing self-esteem. The quality of one’s social relationships may intersect with education to influence self-esteem in a resource multiplication pattern because people with higher education will be better able to take advantage of high-quality relationships due to greater human capital (Leoni, 2023). Consequently, relationship quality would be more strongly associated with self-esteem for those with higher levels of education. Alternatively, relationship quality may intersect with education in a resource substitution pattern because people with low education will depend more on relationship quality for self-esteem due to the lack of alternative resources.

Similarly, path F in Figure 1 demonstrates the interaction between gender and quality of social relationships on self-esteem. In a resource multiplication pattern, one’s quality of social relationships will have stronger benefits for the self-esteem of men because men are accorded more status and prestige in society (Ridgeway, 2011), greater wealth in later-life (Ruel & Hauser, 2013), and less time in caretaking roles (Carr & Utz, 2020), all of which may provide men with more free time and resources to better harness the benefits of quality social relationships. Conversely, a resource substitution pattern, in which women benefit more from the quality of social relationships, may occur because women are more likely to have emotionally intimate relationships which provide social support and improve well-being (Rosenfield & Mouzon, 2013).

## Methods

### Data

Data are derived from the Caregiving, Aging, and Financial Experiences (CAFE) study, a national survey intended to examine social conditions and well-being among older Canadians. Data were gathered by the study authors in cooperation with the Angus Reid Forum (ARF), a Canadian national survey research firm that maintains an ongoing national panel of Canadian respondents from which nationally representative samples can be drawn. The ARF administered the CAFE survey to their panel with the intention of drawing a representative sample of Canadians aged 65–85. Additionally, following data collection, the research team was provided with statistical weights according to the age, gender, and region to ensure a nationally representative sample of older Canadians. These weights were based on most recently available population characteristics from census data provided by Statistics Canada. This weight is applied in all analyses. The CAFE survey was gathered in late September and early October of 2021 as an online survey conducted of 4010 Canadians between age 65 and 85. The response rate was 56%. The final analytic sample for the main analyses with listwise deletion (which refers to the practice of removing individuals with missing data on any variable in the analysis) is 3972 and, as this is less than a 1% reduction, bias due to listwise deletion is minimal.

### Focal Measures

*Self-esteem* was measured using five items adapted from the Rosenberg Self-Esteem Scale, with the five-item version of this scale validated by Monteiro et al. (2022). An exploratory factor analysis of these items indicated one factor with an eigenvalue above 1 that accounted for over 53% of the variance in the items, with all loadings above 0.50. Cronbach’s alpha was 0.84. All responses were coded so that higher values indicate greater self-esteem, and the measure is based on the mean for individuals who responded to at least three of the five items.

*Education* was measured as a set of dichotomous indicators, in which those with post-high school experience, those who graduated from trade school, and those with an undergraduate university degree or greater were compared to those with a high school diploma or less.

*Gender* was measured according to respondent’s self-identified gender, with men coded as 0 and women coded as 1.

*Quality of social relationships* was measured using five positive and negative quality of social relationships items that were adapted from the National Social Life, Health, and Aging Project. An exploratory factor analysis of these items indicated one factor with an eigenvalue above 1 (with the next eigenvalue far below 1) that accounted for over 34% of the variance in the items, with all loadings at approximately 0.50 or above. Cronbach’s Alpha was 0.71. All items were coded so that higher values reflect better quality.

### Covariates

We control for age, visible minority status, marital status, retirement status, and frequency of in-person visits in order to rule out the influence of alternative social statuses and social contact. Age was measured in years. A common approach to race in Canadian research is a general “visible minority” category (Little, 2016), and in keeping with this approach, visible minority status was a dichotomous variable based on the question, “Would you say you are a member of a visible minority here in Canada (in terms of your ethnicity/race)?” with affirmative answers coded as 1. Marital (0 = married and 1 = non-married) and retirement status (0 = not retired and 1 = retired) were dichotomous variables. We also control for frequency of social contact (1 = Never to 6 = Once a day or more) to rule out differences in social network integration that may influence both the quality of social relationships and self-esteem. Table 1 shows descriptives for all study measures.

**Table 1.** Descriptive Statistics.

	Response Scale	Men	Women	Overall
Self-esteem^a^	1–5	4.033	3.922	3.977***
		(0.685)	(0.721)	(0.705)
Education^b^				
High school	0/1	0.137	0.154	0.155
Post-high school experience	0/1	0.251	0.255	0.253
Trade school	0/1	0.208	0.215	0.212
University graduate	0/1	0.403	0.377	0.390
Quality of social relationships^a^	1–5	3.792	3.781	3.787
		(0.602)	(0.651)	(0.627)
Age^a^	65–85	71.815	71.667	71.740
		(5.082)	(4.966)	(5.024)
Visible minority status^b^	0/1			
No		0.937	0.945	0.941
Yes		0.063	0.055	0.059
Marital status^b^	0/1			
Not married		0.334	0.546	0.441***
Married		0.667	0.454	0.559
Retirement status^b^	0/1			
Not retired		0.181	0.142	0.161**
Retired		0.819	0.858	0.839
Frequency of social contact	1–6	3.708	3.798	3.753*
		(1.322)	(1.307)	(1.315)

*N* = 3972. Descriptives are unweighted. Standard deviations are listed in parentheses. *p < .05; **p < .01; ***p < .001. Men are 49.67% of the sample, and women are 50.33% of the sample.

^a^Mean (standard deviation) and *t* test are reported.

^b^Proportion and chi-square test are reported.

### Plan of Analysis

The main analyses utilize a series of multiple regression models based on ordinary least-squares estimation. First, how education, gender, and relationship quality are independently associated with self-esteem is estimated. The second model introduces controls for age, visible minority status, marital status, retirement status, and frequency of social contact. The third model tests an interaction between education and gender, which examines whether the association between education and self-esteem differs between men and women. The fourth model examines an interaction between education and the quality of social relationships. An additional model incorporates an interaction between gender and relationship quality. Together, these two final models test whether the association between relationship quality and self-esteem differs by educational attainment and gender.

## Results

Table 2 reports the regression results. Model 1 indicates that those with post-high school experience (*b* = 0.077, *p* < .05), trade school education (*b* = 0.085, *p* < .05), and those with a university degree (*b* = 0.145, *p* < .001) have significantly higher levels of self-esteem than those with a high school education. Women have significantly lower levels of self-esteem than men (*b* = −0.101, *p* < .001). Additionally, Model 1 shows that quality of social relationships has a statistically significant positive association with self-esteem (*b* = 0.469, *p* < .001). When controls for age, visible minority status, marital status, retirement status, and frequency of in-person visits are introduced in Model 2, two of the three education categories are reduced to non-significance. Those with a university degree continue to have significantly higher levels of self-esteem than those with a high school education (*b* = 0.124, *p* < .001), and women have significantly lower levels of self-esteem than men (*b* = −0.099, *p* < .001). In addition, the quality of social relationships has a statistically significant positive association with self-esteem (*b* = 0.425, *p* < .001).

**Table 2.** Regression Results.

	Model 1			Model 2			Model 3			Model 4			Model 5		
	*B*	SE	*p*	*b*	SE	*p*	*b*	SE	*p*	*b*	SE	*p*	*b*	SE	*p*
Focal predictors															
Education															
Post-high school	0.077	0.036	*	0.059	0.035		0.077	0.052		0.106	0.227		0.066	0.035	
Trade school	0.085	0.037	*	0.068	0.037		0.119	0.052	*	0.144	0.236		0.072	0.037	
University graduate	0.145	0.034	***	0.124	0.033	***	0.192	0.048	***	0.307	0.218		0.128	0.033	***
Gender															
Women	−0.101	0.022	***	−0.099	0.022	***	−0.022	0.057		−0.099	0.022	***	−0.577	0.143	***
Quality of social relationships	0.469	0.018	***	0.425	0.019	***	0.425	0.019	***	0.450	0.045	***	0.354	0.027	***
Control variables															
Age				0.006	0.002	**	0.006	0.002	**	0.006	0.002	**	0.006	0.002	**
Visible minority status				0.042	0.049		0.041	0.049		0.041	0.049		0.040	0.049	
Marital status				−0.050	0.022	*	−0.050	0.022	*	−0.049	0.022	*	−0.051	0.022	*
Retirement status				−0.024	0.029		−0.024	0.029		−0.023	0.029		−0.023	0.029	
Frequency of social contact				0.083	0.009	***	0.084	0.009	***	0.083	0.009	***	0.083	0.009	***
Moderating effects 2-term															
Education*Gender															
Post-high School*Women							−0.032	0.071							
Trade school*Women							−0.092	0.073							
University graduate*Women							−0.126	0.067							
Quality of social relationships*Education															
QoSL*Post-high school										−0.012	0.056				
QoSL*Trade school										−0.020	0.059				
QoSL*University graduate										−0.048	0.054				
Quality of social relationships*Women													0.126	0.036	**
															
Intercept	1.621	0.179	***	1.621	0.179	***	1.578	0.177	***	1.525	0.239	***	1.879	0.190	***
R2	0.209		0.209	0.210		0.209	0.212								

*p ≤ .05; **p ≤ .01; ***p ≤ .001 (two-tailed tests). *N* = 3972.

Model 3 tests interactions between the categories of education and gender, but none of these interactions are significant. Model 4 then tests interactions between the categories of education and quality of social relationships, but none of these interactions are significant, either. Thus, individuals with a high level of education have significantly higher levels of self-esteem, but this advantage does not differ by gender. The positive association between quality of social relationships and self-esteem also does not differ significantly by levels of education. Associations between education and self-esteem therefore do not conform to a resource multiplication *or* a resource substitution pattern for intersections with gender or quality of social relationships.

**Figure 2.**
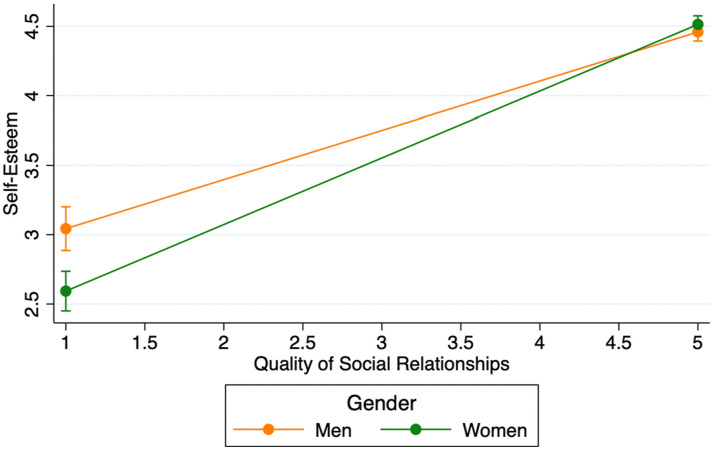
Associations between quality of social relationships and self-esteem for men and women.

Model 5 includes an interaction between gender and quality of social relationships. This interaction is significant (*p* < .01), indicating that the association between relationship quality and self-esteem does differ significantly between men and women. Figure 2 explicates the meaning of this interaction. This figure shows that the association between quality of social relationships is positive for both men and women, but this association is stronger for women than men (men: *b* = 0.354, *p* < .001; women: = 0.480, *p* < .001). That there is a stronger association between quality of social relationships and self-esteem for women than men conforms to a resource substitution pattern. Moreover, as this figure also shows, there is a significant gender difference in self-esteem at low levels of quality of social relationships (*b* = −.450, *p* < .001), but this difference is not significant high levels of quality of social relationships (*b* = .054, *p* > .10). Thus, the stronger association between quality of social relationships and self-esteem for women means that, at high levels of quality of social relationships, the gender difference in self-esteem is negated.

## Discussion

Self-esteem is an important resource for the mental and physical health of older adults (Krause, 2016). A stress process perspective suggests that this resource is likely to be shaped by fundamental social statuses and the quality of one’s social relationships, as well as their intersection (Pearlin & Bierman, 2013). Yet, the role of these factors in self-esteem has generally not been examined recently in Canadian national studies focusing on older adults, even though gender roles and the role of education in SES have changed substantially over time (Mirowsky & Ross, 2007). Moreover, little research has examined how gender, education, and social resources intersect in shaping self-esteem in later-life, suggesting a need to better understand how social statuses and relationships may cohere to shape self-esteem among older adults.

Our analyses show mixed results for education and self-esteem. With background controls, only older adults with higher education retained an advantaged position in self-esteem. Moreover, we observed no evidence of resource multiplication or substitution, as indicated by a lack of intersection with both gender and quality of social relationships. Educational attainment is typically established relatively early in adulthood. That people with a university degree continue to show higher levels of self-esteem into later-life suggests that there is a robust advantage across the life-course for one’s self-esteem due to high levels of education. Furthermore, that we do not observe a resource multiplication or substitution pattern also speaks to the potency of education for self-esteem—a high level of educational attainment appears to be a master status that determines self-esteem irrespective of other markers of social standing. Attainment of a university degree likely benefits self-esteem because individuals are able to accumulate knowledge, skills, and social ties, as well as jobs of higher prestige and earning potential. These accomplishments will not just lead to higher self-esteem based on social comparisons and self-attributions. Additionally, these accomplishments will tend to improve how others view such individuals in later-life (von Soest et al., 2018) which will bolster self-esteem according to the reflected appraisals principle. A consideration of these more proximal factors in the causal path between education and self-esteem would likely show more clearly how a relatively high level of education benefits self-esteem in later-life. We therefore suggest that an important direction for future research is to trace out the pathways by which educational attainment influences self-esteem in later-life using contemporaneous samples of older adults.

A lack of resource multiplication or substitution in education is also of interest in the context of gender differences in self-esteem. These results suggest that obtaining a high level of education may help women *offset* gendered disadvantages in self-esteem in later-life, but high educational attainment will not negate these gender disadvantages. Even with a high level of education, women still appear to retain a gender disadvantage in self-esteem compared to men in later-life. This may be due to economic dependence, limitations with paid employment, and inequity in the division of household labor (Ross & Mirowsky, 2006), which according to the principle of self-attributions, influences self-esteem as women may feel a lack of personal effectiveness and inadequacy in accomplishing their goals (Maclean & Hill, 2015). Notably, the intersection of gender and education in predicting self-esteem has not generally been recently examined in large-scale studies of older adults, but these results are different from previous analyses based on older surveys that indicated a gender difference in the consequences of education for self-esteem (Schieman, 2002), as well as research showing that education and gender intersect in shaping symptoms of depression (Ross & Mirowsky, 2006). Differences with previous findings and ours likely occurred in part because Schieman’s (2002) study focused on adults aged 18–55, in which the experience of older aged adults was not examined. This pattern of findings therefore suggests that studies of older adults may show different associations than individuals earlier in the life course. More centrally, though, this pattern of findings across studies underscores not only that self-esteem may differ as an outcome from related mental health outcomes, but also that these associations likely changed as gender and educational norms have evolved over time.

The quality of social relationships was also shown to be associated with higher self-esteem in older adults. This finding supports the general sociological contention that self-esteem is a social product (Serpe et al., 2019), as strong relationships with others likely provide a key source for a positive sense of one’s self. Maintaining high-quality social relationships also appears to be especially important for women, as the quality of social relationships was more strongly associated with self-esteem for women than men. Consequently, gender differences in self-esteem were negated at high levels of quality of social relationships. The gender difference in the association between the quality of social relationships and self-esteem likely occurs in part because women tend to feel more open in their social relationships (Felmlee et al., 2012) and are therefore better able to gain the compensation of supportive and rewarding social exchanges. Consequently, programs which focus on helping individuals to cultivate and maintain high-quality social relationships in later-life are not only likely to help older adults maintain self-esteem, but also build greater self-esteem. Furthermore, such programmatic efforts are also likely to help address the gender gap in self-esteem among older adults.

There are, however, limitations to this study that should be noted. First, as this study is focused on Canadian older adults, it is possible that nations with stronger support systems for older adults may show weaker social status differences in self-esteem. Second, participants were surveyed during the COVID-19 pandemic, which may have skewed perceptions regarding various aspects of social relationships due to restrictions surrounding social contact. However, the consequences of the quality of social relationships for self-esteem may be even stronger when older adults are freer to interact with members of their social network. Finally, analyses of longitudinal data over time are likely to better show how social statuses and social relationships shape self-esteem well into later-life. We therefore recommend that large-scale surveys of older adults more routinely include measures of self-esteem.

## Conclusion

The current research presents an important contribution to the study of social stratification and self-esteem by showing how social statuses and relationships can continue to contribute to self-esteem well into later-life. Disadvantages in social statuses demarcate substantial challenges to individuals’ abilities to foster a positive sense of self, with a reduced sense of self-worth likely to negatively impact physical and mental health. However, maintaining high-quality relationships can help to offset, and in the case of gender, negate these disadvantages. Self-esteem continues to be key to individual well-being, and more contemporaneous attention is needed to the dynamics that shape this psychological resource in later-life.

## Note

1. Recent Statistics Canada census data shows that individuals who identify as transgender or non-binary are relatively rare among older adults, comprising less than a half a percent of the population in this age range (Statistics Canada, 2022). Not surprisingly, then, all respondents in our study identified as men or women, even with an additional option to self-describe. In this research, we therefore not only examine gender differences in terms of between men and women, but also acknowledge the importance of examining self-esteem in the context of the aging experience for transgender and non-binary individuals, especially as younger cohorts approach middle and later-life.
